# Lipoprotein (a) in the Development and Progression of Diabetic Retinopathy: A Systematic Review and Meta-Analysis

**DOI:** 10.3390/medicina61071137

**Published:** 2025-06-24

**Authors:** Stamatios Lampsas, Vaia Lambadiari, Chrysa Agapitou, Aikaterini Lampsa, Evangelos Oikonomou, Gerasimos Siasos, Irini Chatziralli

**Affiliations:** 1 2nd Department of Ophthalmology, Attikon University Hospital, Medical School, National and Kapodistrian University of Athens, 11527 Athens, Greece; lampsas.stam@gmail.com (S.L.); chr.agapitou@gmail.com (C.A.); 2 Diabetes Center, 2nd Department of Internal Medicine, Attikon University Hospital, Medical School, National and Kapodistrian University of Athens, 11527 Athens, Greece; vlambadiari@gmail.com (V.L.); katerinalam2000@hotmail.com (A.L.); 3 3rd Department of Cardiology, Thoracic Diseases General Hospital Sotiria, Medical School, National and Kapodistrian University of Athens, 11527 Athens, Greece; boikono@gmail.com (E.O.); ger_sias@hotmail.com (G.S.); 4 Cardiovascular Division, Harvard Medical School, Brigham and Women’s Hospital, Boston, MA 02115, USA

**Keywords:** lipoprotein(a), diabetic retinopathy, microvasculature, atherosclerosis, progression

## Abstract

*Background and Objectives*: Diabetic retinopathy (DR) is a significant complication of Diabetes Mellitus. Several studies have indicated Lipoprotein (a) [Lp(a)] plays a role in atherosclerotic alterations. *Materials and Methods*: This meta-analysis/systematic review aims to investigate the connection between Lp(a) and DR. All relevant studies indexed in PubMed and Scopus from up to January 2025 were included. A total of 29 studies (7007 subjects) were included, and the results were synthesized according to the PRISMA Guidelines. The results are presented as standardized mean differences (SMDs) with 95% confidence intervals (CIs) derived using random effects models. *Results*: The mean age of the included subjects was 52.6 ± 9.4 years, with 52.5% being male. The primary analysis included 25 observational studies involving a total of 6291 subjects (2770 patients with DR vs. 3521 controls). Notably, Lp(a) levels were significantly higher in patients with DR compared to those in controls, with an SMD of 0.85 (95% CI: 0.48–1.22; *p* < 0.001, I^2^ = 98%). Interestingly, a secondary analysis of the patients with Proliferative Diabetic Retinopathy (PDR) and Non-Proliferative Diabetic Retinopathy (NPDR) yielded an SMD of 0.28 (95% CI: 0.09–0.47; *p* = 0.004, I^2^ = 97%) between the two compared groups. In this analysis, a total of 1066 patients (465 PDR patients vs. 601 NPDR patients) were included. *Conclusions*: Elevated Lp(a) levels may have a compelling relationship with the development and progression of DR based on the evidence analyzed.

## 1. Introduction

Diabetic Retinopathy (DR), a microangiopathy of diabetes mellitus (DM) with a prevalence rising alongside the global increase in diabetes cases, refers to damage to small blood vessels due to chronic-DM-induced hyperglycemia. The global prevalence of DR in patients with type 2 DM is estimated to be 30–40%, with 5–15% of cases classified as vision-threatening [1,2]. Several clinical trials and epidemiological studies have identified DM duration, hypertension, and abnormal blood-glucose metabolism as the main risk factors for DR, with dyslipidemia, chronic low-grade inflammation, and obesity also playing a crucial role in the onset and development of DM-related complications [3].

Lipoprotein(a) [Lp(a)] is an emerging atherosclerotic biomarker and a unique lipid particle structurally similar to low-density lipoprotein (LDL) to which an additional glycoprotein protein called apolipoprotein (a) is attached [4,5]. Lp(a)’s plasma concentration remains relatively constant throughout an individual’s life, as this concentration is primarily determined by genetic factors [6]. Elevated plasma Lp(a) levels have been implicated in various microvascular complications, including endothelial dysfunction, oxidative stress, and inflammatory responses, which are relevant to the progression of DR [7,8]. Particularly, Lp(a) has been associated with reduced nitric oxide bioavailability that leads to increased quantities of reactive oxygen species (ROS), resulting in early capillary retinal endothelial injury and blood–retinal barrier disruption [9]. Furthermore, a pivotal role is played by Lp(a) in the expression of a plethora of pro-inflammatory cytokines (IL-6 and TNF-α) and adhesion molecules [ICAM-1 (Intercellular Adhesion Molecule-1) and VCAM-1 (Vascular Cell Adhesion Molecule-1)] that trigger leukostasis and chronic low-grade retinal inflammation, amplifying endothelial loss of integrity, which is observed in early non-proliferative DR [7]. However, the role Lp(a) plays in the progression of retinopathy may be catalytic because of its prothrombotic and vasoconstrictive actions due to its structural homology to plasminogen, which worsens retinal ischemia. The production of vascular endothelial growth factor (VEGF) and the promotion of abnormal neovascularization result in the progression to Proliferative Diabetic Retinopathy (PDR), which is exacerbated since Lp(a) amplifies oxidative stress (OS) in the retinal microvasculature, also impairing retinal pericyte viability and leading to structural changes in the vitreoretinal interface, thereby potentially progressing retinal microvascular alterations [4]. However, the relationship between Lp(a) and DR has been inconsistently investigated, with studies showing conflicting results: some studies show a positive association, while others show no significant link [10,11].

This meta-analysis/systematic review aims to consolidate current evidence regarding the role of elevated Lp(a) levels in the development and progression of DR but also shed light on the underlying mechanisms contributing to retinopathy in patients with DM.

## 2. Materials and Methods

### 2.1. Literature Search

We adhered to the methodological framework provided by the Preferred Reporting Items for Systematic Reviews and Meta-Analysis (PRISMA) guidelines when conducting this study [12]. The protocol has been registered in PROSPERO (ID: CRD42024596117). We systematically searched the PubMed and Scopus databases up to 22 January 2025 for articles examining the role of plasma Lp(a) in Diabetic Retinopathy. The search terms were applied to meet these criteria, using the following search query: “Lipoprotein(a)” OR “Lp(a)” OR “Lipoprotein-a”. The following keywords were utilized to identify the relevant outcome variables of interest: “Diabetic Retinopathy” OR “DR” OR “Diabetes Mellitus AND “Retinopathy” OR “Diabetes Retinopathy”. Two researchers independently evaluated the articles for eligibility using predefined selection criteria. Any discrepancies were managed through repeated assessments and consensus among the authors. Database searches were conducted again prior to the final analysis, and any relevant additional studies were retrieved for inclusion. Reference lists of the retrieved articles were also reviewed using the “snowballing” technique to identify additional publications.

### 2.2. Study Selection and Data Extraction

All original observational studies, including cohort, cross-sectional, and case–control designs, were included based on the following study criteria. (i) All studies involved human adults (aged 18 years or older), both males and females. (ii) Only peer-reviewed articles and (iii) articles written in English were included. (iv) The studies addressed soluble plasma concentrations of Lp(a) in patients with DR. Moreover, inclusion criteria were precisely established using the PECOS framework to ensure clarity and precision in this systematic review and meta-analysis: (i) participants (P)—patients with diabetes, both with and without DR; (ii) exposure (E)—elevated levels of Lp(a); (iii) comparator (C)—patients with diabetes without DR (comparison group with normal or lower levels of Lp(a); and outcome (O)—DR in relation to levels of Lp(a), assessing whether higher Lp(a) levels are associated with an increased risk or severity of DR. The following research was excluded from the analysis: (i) all reviews, systematic reviews, and meta-analyses; (ii) non-English language studies; (iii) animal studies; (iv) studies where the levels of plasma Lp(a) were unavailable; and (v) studies in which non-diabetic subjects were included in the control group. Following the completion of screening, the following data were extracted from the included studies by two independent investigators using a predefined data collection form: (i) first author’s name and the year of publication; (ii) study design; (iii) sample size; (iv) mean age and male sex ratio for the whole sample; and (v) the groups compared in the included studies. Authors were contacted by email to obtain information unavailable in the published articles. Screening was conducted following the removal of duplicated citations through the help of Rayyan systematic review and Zotero software (Version: 7.0.12).

### 2.3. Quality Assessment

The quality assessment and risk-of-bias assessment tools provided with the Newcastle–Ottawa Scale (NOS) were applied by two researchers to the 29 studies meeting the eligibility criteria; these tools were adapted for case–control, cross-sectional, and cohort studies [13]. Their evaluation was based on three main domains: how the study groups were selected, the groups’ comparability, and the ascertainment of either the exposure or outcome of interest. Scores of 0–3 for low quality, 4–6 for moderate quality, and 7–9 (or 7–10 for cross-sectional studies) for high quality were allocated. The highest possible scores is 9 for case–control and cohort studies and 10 for cross-sectional studies. The overall quality was found to be high for 19 studies (66%) and moderate for the remaining 10 (34%). Cohort studies showed an average score of 7.5 out of 9, case–control studies exhibited an average score of 8 out of 9, and cross-sectional studies had an average score of 6.4 out of 10. A detailed report is included in Supplementary Table S1.

### 2.4. Statistical Analysis

Data collection involved both direct extraction and estimation, applying Wan et al.’s approach to convert median and interquartile range (IQR) values into means and standard deviations (SDs) [14]. Forest plots were employed to illustrate the results. To evaluate the differences, the standardized mean differences (SMDs) between the groups and their 95% confidence intervals (CIs) were calculated using random-effects models. Statistical heterogeneity was evaluated using the Q statistic, derived from the χ^2^ test (with a significance threshold of *p* = 0.05), while the I^2^ statistic was used to quantify the variance due to between-study heterogeneity. I^2^ values higher than 75% indicated substantial heterogeneity, 50–75% signified moderate heterogeneity, and values below 50% represented low heterogeneity. In case of high heterogeneity, outlying and highly influential studies were identified (using the leave-one-out method), and the sensitivity analysis was repeated after these studies were excluded [15]. All statistical analyses were conducted using RevMan 5.4 software (The Cochrane Collaboration, Oxford, UK). The significance threshold was set to 0.05.

## 3. Results

### 3.1. Search Results

**Figure 1:**
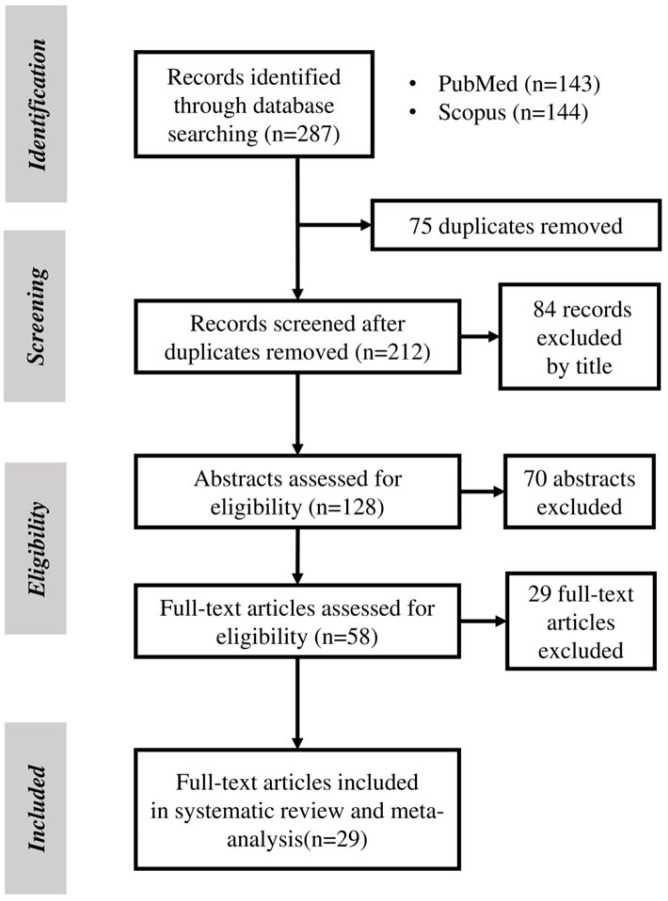
PRISMA flowchart for study selection.

Out of the initial 287 studies, 29 were finally included in the systematic review and meta-analysis after exclusion and inclusion criteria were applied (Figure 1). A total of *n* = 7007 subjects were included, with an average mean age of 52.6 ± 9.4 years and a male sex prevalence of 52.5%. Most of the studies (79.3%) were cross-sectional (*n* = 23), while 17.8% adopted a case–control design (*n* = 4), and 6.9% employed a cohort design (*n* = 2) (Table 1) [10,11,16,17,18,19,20,21,22,23,24,25,26,27,28,29,30,31,32,33,34,35,36,37,38,39,40,41,42].

### 3.2. Quantitative Synthesis

#### 3.2.1. Comparison of Lp(a) Levels Between Subjects with and Without DR

**Figure 2:**
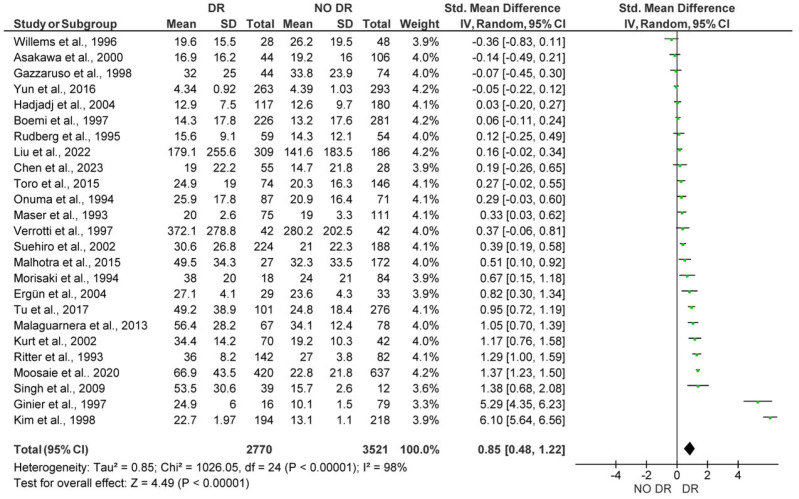
Forest plot showing the pooled standardized mean difference (SMD) in lipoprotein(a) [Lp(a)] levels between patients with Diabetic Retinopathy (DR) and diabetic patients without Diabetic Retinopathy (NO DR). Random-effects model was applied. SD, standard deviation; CI, confidence interval.

**Figure 3:**
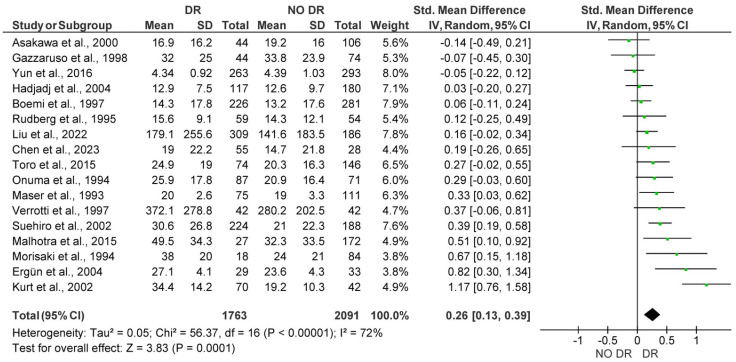
Forest plot showing the pooled standardized mean difference (SMD) of the sensitivity analysis after removing outlier studies for lipoprotein(a) [Lp(a)] levels between patients with Diabetic Retinopathy (DR) and diabetic patients without Diabetic Retinopathy (NO DR). Random-effects model was applied. SD, standard deviation; CI, confidence interval.

The 25 studies analyzed in this analysis included a total of 6291 subjects (2770 patients with DR vs. 3521 control subjects). The meta-analysis showed significantly higher Lp(a) levels for patients with DR, with a pooled SMD of 0.85 (95% CI: 0.48–1.22; *p* < 0.001). However, substantial heterogeneity (I^2^ = 98%) was detected among studies (Figure 2). After funnel plot evaluation, the sensitivity analysis confirmed the results showing significantly elevated Lp(a) concentrations in patients with DR, with a pooled SMD of 0.26 (95% CI: 0.13–0.39; *p* < 0.001), with moderate heterogeneity (I^2^ = 72%) among studies. In the sensitivity analysis, a total of 3084 subjects were included (1763 patients with DR vs. 2091 control subjects) after eight outlier studies were removed (Figure 3).

#### 3.2.2. Lp(a) Levels in Subjects with Non-Proliferative Diabetic Retinopathy (NPDR) vs. Proliferative Diabetic Retinopathy (PDR)

**Figure 4:**
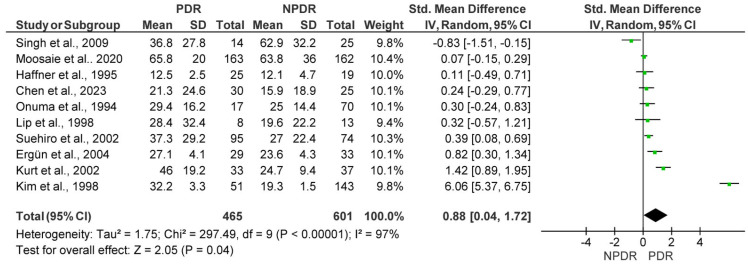
Forest plot showing the pooled standardized mean difference (SMD) in lipoprotein(a) [Lp(a)] levels between patients with Proliferative Diabetic Retinopathy (PDR) and those with Non-Proliferative Diabetic Retinopathy (NPDR). Random-effects model was applied. SD, standard deviation; CI, confidence interval.

**Figure 5:**
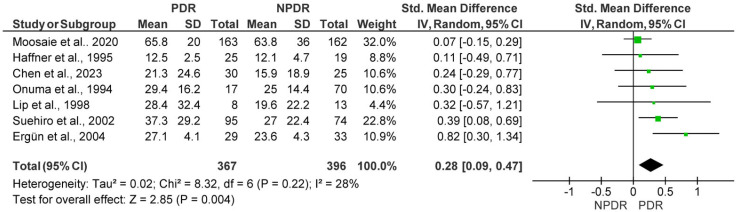
Forest plot showing the pooled standardized mean difference (SMD) of the sensitivity analysis, after outlier studies were removed, for lipoprotein(a) [Lp(a)] between patients with Proliferative Diabetic Retinopathy (PDR) and those with Non-Proliferative Diabetic Retinopathy (NPDR). Random-effects model was applied. SD, standard deviation; CI, confidence interval.

Among the studies included in the meta-analysis, 10 compared Lp(a) levels between patients with PDR and those with NPDR, amounting to a total of 1066 patients (465 PDR patients vs. 601 NPDR patients). The meta-analysis demonstrated significantly higher Lp(a) levels in PDR patients, with a pooled SMD of 0.88 (95% CI: 0.04–1.72; *p* = 0.04). The heterogeneity among the included studies was substantial (I^2^ = 97%) (Figure 4). The sensitivity analysis confirmed the results showing higher Lp(a) in patients with PDR, showing a pooled SMD of 0.28 (95% CI: 0.09–0.47; *p* = 0.004). In the sensitivity analysis, three outlier studies were removed after funnel plot assessment, including seven studies with a total of 763 patients (367 PDR vs. 396 NPDR patients), and there was low heterogeneity among studies (I^2^ = 28%) (Figure 5).

## 4. Discussion

The aim of this systematic review and meta-analysis was to elucidate the association between plasma Lp(a) levels and the development and progression of DR in patients with diabetes. The current study, based on a comprehensive analysis of 29 studies with a total of 7007 subjects, provides a shred of robust evidence of the potential atherogenic role of Lp(a) in microvascular complications, including DR.

The primary analysis demonstrated a substantial increase in Lp(a) levels in patients with DR compared to those in individuals without DR. The results of this analysis support the hypothesis that Lp(a) plays a crucial role in the pathogenesis of DR, potentially through mechanisms related to endothelial dysfunction, oxidative stress, and inflammation. Elevated Lp(a) levels are highly associated with multiple pro-inflammatory effects, being a major carrier of oxidized phospholipids (OxPL) in the bloodstream and having an important role in the upregulation of pro-inflammatory cytokines, such as interleukin-1β (IL-1β), IL-6, and tumor necrosis factor-α (TNF-α) in various cell types [43,44]. Moreover, Lp(a) seems to contribute to the accumulation of various matrix components, triggering the recruitment and activation of monocytes and macrophages, which leads to endothelial dysfunction, stimulates smooth muscle cell (SMC) growth, and amplifies localized inflammation [45].

Furthermore, studies have revealed that Lp(a) triggers OS in monocytes, leading to DNA damage and mutations that further increase inflammatory gene expression [46]. These mechanisms are consistent with previous studies that have implicated Lp(a) in other vascular complications since Lp(a) seems to affect retinal microvasculature, impairing vascular integrity and promoting the progression of DR [7]. Experimental evidence supports this: studies involving human retinal endothelial cells have revealed that Lp(a) exposure results in heightened oxidative stress, impaired mitochondrial membrane potential, and upregulation of VEGF, a key regulator for PDR [47]. Also, models of transgenic mice expressing human Lp(a) have demonstrated greater retinal vascular permeability and leukocyte adhesion, particularly during hyperglycemia, indicating that Lp(a) contributes to the worsening of diabetes-induced microvascular injuries [48]. Additionally, Lp(a)-induced OS in monocytes results in mitochondrial DNA damage and mutation, enhancing inflammatory signaling and potentially establishing a vicious cycle of chronic low-grade inflammation and microvascular dysfunction, pathophysiological alterations that trigger the early DR finding in patients with DM [49].

Additionally, the analysis demonstrated a significant association between higher Lp(a) levels and the severity of DR, as shown by the comparison between patients with NPDR and those with PDR. This gradient suggests that Lp(a) is not only related to the development of the disease but also the progression to more advanced stages of the disease since PDR is associated with more severe microvascular damage [50]. Notably, Deraza et al. reported that Lp(a) is significantly positively associated with central macular thickness, and higher Lp(a) levels, namely, 11.34 ng/mL, showed accuracy in predicting central macular edema (CME), indicating that Lp(a) can be used as a potential marker for diabetic maculopathy detection [16]. Another mechanism in which Lp(a) is highly involved is vascular remodeling, which is related to DR severity [7]. Lp(a) is known to enhance the deposition of lipids and other inflammatory markers within the arterial wall, contributing to plaque formation and the loss of vascular integrity/permeability [51]. It has been noted that Lp(a) enhances endothelial cell contraction and permeability through a Rho/Rho kinase-dependent signaling pathway [52]. Notably, experimental studies conducted on both animal models and in vitro endothelial cell cultures have shown that transgenic mice overexpressing apolipoprotein(a) exhibit enhanced retinal vascular leakage and pericyte dropout under hyperglycemic conditions [53].

High plasma Lp(a) levels have also been associated with higher oxidative in endothelial and retinal cells, causing mitochondrial damage and metabolic impairment [54]. OS triggered by high Lp(a) levels is closely linked to elevated VEGF synthesis, promoting neovascularization and retinal hemorrhage [55]. VEGF plays a key role in pathological angiogenesis and increased vascular permeability, both of which are central to the progression of diabetic retinopathy, particularly in its proliferative form [56]. Moreover, high Lp(a) levels combined with hyperglycemia aggravate the deposition of inflammatory extracellular matrix proteins and enhance matrix metalloproteinase (MMP) activity, leading to a substantial rise in retinal VEGF levels [57]. This multifaced influence on microvasculature reveals the pivotal role of Lp(a) in microvascular complications not only in patients with DM but also in patients without DM (Table 2).

Although Lp(a) has been recognized as an independent risk factor for atherosclerotic cardiovascular disease (ASCVD) by the European Society of Cardiology (ESC) and the American College of Cardiology (ACC), emerging data reveal notable differences in Lp(a) levels among various racial and ethnic populations [58]. However, most guidelines agree that a Lp(a) concentration > 50 mg/dL (or >125 nmol/L) is clinically significant and associated with increased ASCVD risk and DR-related complications [59,60,61]. Interestingly, populations of African descent, particularly Black people of African descent, have significantly higher Lp(a) concentrations, on average, than Caucasians, Hispanics, or East Asians [62]. Moreover, South Asians tend to have higher Lp(a) levels than Caucasians and East Asians, with East Asian groups (Han Chinese and Japanese) generally exhibiting significantly lower Lp(a) concentrations than other races [62].

Therefore, several lipid-lowering therapies with modest or incidental effects on Lp(a) have already been investigated. Particularly, Niacin has been shown to reduce Lp(a) levels by approximately 20–30%, but its use is limited due to its poor tolerability and a lack of cardiovascular benefits in large trials [63,64]. Statins, while highly effective in lowering LDL levels and overall cardiovascular risk, have minimal to no effect on Lp(a) and may slightly increase its levels [65]. Ezetimibe, another LDL-lowering agent, has a negligible impact on Lp(a) concentrations [66]. PCSK9 inhibitors, such as evolocumab and alirocumab, also reduce Lp(a) levels by about 20–30%, with a demonstrated ability to reduce cardiovascular risk, though the Lp(a)-lowering mechanism remains unclear [67].

The latest approaches to treatment focus on reducing levels of Lp(a) by disrupting its production in the liver using RNA-directed agents. Endogenous RNA interference has revealed key regulators of protein expression and opened the door to organ-targeted RNA-based therapies. Specifically, Pelacarsen, a GalNAc-conjugated antisense drug targeting apo(a), lowers Lp(a) levels by 66–92%, with good tolerability and few mild injection-site reactions [68]. Olpasiran, a small interfering RNA (siRNA) agent, by reducing apo(a) formation, achieved an up to 95% reduction in Lp (a) levels [69]. Another 19-mer siRNA molecule attached to a tri-antennary GalNAc structure, Zerlasiran, has also achieved an up to 95% reduction in Lp (a) levels [70]. Finally, Lepodisiran, a Dicer-substrate siRNA with a tetraloop and three GalNAc conjugates, was well tolerated and produced reductions of up to 95% in plasma Lp (a) levels [71]. Interestingly, Muvalaplin is the only targeted oral therapeutic to produce robust reductions in Lp(a) levels, with preclinical studies of muvalaplin demonstrating Lp(a) level reductions of up to 65% [72]. These promising RNA-based and oral therapies for lowering Lp(a) are currently in various phases of clinical trials, and future studies are expected to clarify their impact on Lp(a)-related microvascular complications, including diabetic retinopathy (Table 3).

However, the high heterogeneity of the main analyses reflects variability among studies in the association between Lp(a) and DR and the variability in Lp(a) measurement methods [74]. Interestingly, another limitation of this study is the conversion of median and interquartile range (IQR) values to means and standard deviations (SDs), which could have resulted in estimation errors. Moreover, most of the included studies were cross-sectional, limiting the ability to infer causal relationships. Furthermore, variability in Lp(a) assay methods across studies and the absence of standardized units (e.g., mg/dL vs. nmol/L) may have introduced measurement bias. Nevertheless, this study represents a comprehensive and up-to-date systematic review and meta-analysis including a large pooled sample size of 7007 individuals from 29 independent studies.

Despite this study’s compelling findings, several gaps remain. For instance, how Lp(a)-isoform size variability and ethnic-related variations contribute to DR susceptibility remains unclear. Moreover, the cross-sectional design of most of the included studies limits the ability to infer a causal relationship between Lp(a) and DR development and progression. Future researchers should prioritize the performance of prospective longitudinal studies to determine the predictive utility of Lp(a) for the initiation and advancement of diabetic retinopathy treatment across heterogeneous populations. Moreover, targeted interventional trials using emerging therapies for Lp(a) level reduction can mitigate the chronic microvascular and macrovascular Lp(a)-induced alterations, which remain a persistent global challenge.

## 5. Conclusions

This systematic review/meta-analysis provides substantial evidence for the association between elevated plasma Lp(a) levels and the development as well as progression of DR. The higher Lp(a) levels observed in patients with DR compared to those observed in individuals without DR, as well as those in patients with PDR compared to those in individuals with NPDR, indicate that Lp(a) is significantly related to retinal microvascular alterations. Further studies employing standardized Lp(a) measurement methods are needed to validate this causal relationship, shedding light for targeted Lp(a) treatment strategies in the future.

## Figures and Tables

**Table 1:** Characteristics and findings of the included studies.

Study	Study Design	Sample Size	Age (Yrs)	Sex (Male)	Compared Groups	Key Findings
Deraz et al., 2021 [16]	Case–control	40	52.7 ± 5.2	47.5%	1. NO DM and NO-DR 2. DR	Lp(a) levels of 11.34 ng/mL or more were found to have 95% power in predicting central macular edema in patients with DR and can be used as a marker for its detection. A positive correlation was found between central macular thickness and plasma levels of Lp(a) (*p* < 0.001).
Kurt et al., 2002 [17]	Cross-sectional	112	61.7 ± 6.4	50.9%	1. NO DM and NO DR 2. DM and NO DR 3. NPDR 4. PDR	Lp(a) levels were higher in patients with type 2 DM and either background or proliferative retinopathy compared with those in nondiabetic controls and diabetic patients without retinopathy. A clear relationship was detected between higher lipoprotein (a) levels and progression of retinopathy.
Malhotra et al., 2014 [18]	Cross-sectional	199	61 ± 9	51.%	1. DM and NO DR 2. DR	Patients with DR had significantly higher levels of Lp(a) than those without retinopathy (*p* = 0.004).
Singh et al., 2009 [19]	Cross-sectional	51	N/A	100%	1. DM and NO DR 2. NPDR 3. PDR	Lp(a) levels were elevated in patients with DR Patients with PDR had lower Lp(a) levels than those with NPDR, suggesting that the atherogenic phenotype of dyslipidemia retards the development of severe diabetic retinopathy.
Rudberg et al., 1995 [20]	Cross-sectional	133	15.7 ± 1.6	46.6%	1. DM and NO DR 2. DR	Patients with DR had Lp(a) levels similar to those without DR; no correlation was observed between retinopathy and Lp(a) levels.
Tu et al., 2017 [21]	Cross-sectional	377	58 ± 12.4	52.7%	1. DM and NO DR 2. DR	Patients in the highest-Lp(a)-concentration quartiles had a greater risk of developing vision-threatening DR or DR, regardless of whether glycemic control (HbA1c < 7.0%) was achieved.
Moosaie et al., 2020 [22]	Case–control	1057	56.8 ± 9.7	52.9%	1. DM and NO DR 2. DR 3. NPDR 4. PDR	A positive relationship was detected between Lp(a) levels and the presence of DR, independent of albuminuria, duration of diabetes, and type of diabetes treatment.
Yun et al., 2016 [23]	Cohort	556	54.2 ± 10	42.4%	1. DM and NO DR 2. DR	Lp(a) levels and DR development were independently associated in patients with type 2 DM Patients in the highest Lp(a) quartiles had a higher DR risk.
Malaguarnera et al., 2013 [24]	Cross-sectional	145	66.8 ± 12.4	43.4%	1. DM and NO DR 2. DR	A significant proportion of patients with retinopathy reported notably high levels of plasma Lp(a).
Liu et al., 2022 [25]	Case–control	667	56.3 ± 10.2	50.7%	1. NO DM and NO DR 2. DM and NO DR 3. DR	Plasma Lp(a) levels showed no difference between subjects with DR and controls.
Chandni et al., 2012 [26]	Cross-sectional	144	53.9 ± 10.7	56.9%	1. NO DM and NO DR 2. DR	There was no statistically significant difference in Lp(a) levels among patients with and without diabetic retinopathy.
Morisaki et al., 1994 [27]	Cross-sectional	104	66 ± 10	34%	1. DM and NO DR 2. DR	The group with retinopathy had significantly higher Lp(a) levels than the group without it. Plasma Lp(a) was an independent risk factor for retinopathy in all cases and in the elderly; the incidence of retinopathy was positively correlated with serum Lp(a) levels.
Haffner et al., 1995 [28]	Cross-sectional	70	61.4 ± 2.1	58%	1. DM and NO DR 2. MILD NPDR 3. MODERATE NPDR 4. PDR	No increased prevalence of retinopathy in subjects with higher Lp(a) levels was detected in either younger-onset or older-onset individuals.
Ergün et al., 2004 [29]	Cross-sectional	100	57.5 ± 3.1	33%	1. DM and NO DR 2. DR 3. PDR	The Lp(a) levels were similar in the patients with and without retinopathy. No evidence of a relationship between serum Lp(a) levels and diabetic retinopathy in type 2 DM was detected.
Chopra et al., 2007 [30]	Cross-sectional	200	55.1 (N/A)	41.5%	1. DM and NO DR 2. PDR	The average Lp(a) levels in patients with retinopathy were significantly higher compared to those in the control group. Subjects with PDR had higher Lp(a) levels than those with NPDR.
Onuma et al., 1994 [31]	Cross-sectional	158	58.6 ± 11.4	52.5%	1. DM+ NO DR 2. NPDR 3. PDR	Lp(a) levels in patients with simple retinopathy were significantly higher than in control subjects. Patients with proliferative retinopathy reported significantly higher Lp(a) levels than patients without retinopathy and control subjects.
Willems et al., 1996 [10]	Cross-sectional	106	N/A	61.3%	1. DM and NO DR 2. DR	Lp(a) levels were not significantly increased in patients with retinopathy versus those without retinopathy.
Suehiro et al., 2002 [32]	Cross-sectional	412	57.4 ± 12.5	56.8%	1. NO DM and NO DR 2. DM and NO DR 3. NPDR 4. SEVERE NPDR 5. PDR	Patients with DR, especially those with proliferative retinopathy, had significantly higher serum concentrations of Lp(a) than those without DR.
Verrotti et al., 1997 [33]	Cross-sectional	126	20 ± 5.5	50%	1. NO DM and NO DR 2. DM and NO DR 3. NPDR 4. PDR	Patients with retinopathy did not exhibit any significant differences in serum levels of lipids and Lp(a) when compared with patients without retinopathy and normal subjects. Subjects with PDR were found to have significantly higher Lp(a) compared to NPDR subjects, showing that Lp(a) might play a role in the development of severe retinopathy.
Kim et al., 1998 [11]	Cross-sectional	412	56.8 ± 0.8	N/A	1. DM and NO DR 2. NPDR 3. PDR	The patients with PDR had higher serum Lp(a) levels than those without diabetic retinopathy or with NPDR.
Ritter et al., 1993 [34]	Cross-sectional	224	49.8 (N/A)	56.2%	1. DM and NO DR 2. DR	Proliferative or background retinopathy was associated with significantly higher Lp(a) serum levels.
Boemi et al., 1997 [35]	Cross-sectional	507	54.6 ± 16.6	48.9%	1. DM and NO DR 2. DR	No evidence that Lp(a) is associated with retinopathy was found
Chen et al., 2022 [36]	Case–control	113	53.9 ± 9.4	81.9%	1. NO DM and NO DR 2. DM and NO DR 3. NPDR 4. PDR	No significant difference in Lp(a) levels was found between the compared groups.
Gazzaruso et al., 1998 [37]	Cross-sectional	245	21.9 ± 4.0	55.1%	1. DM and NO DR 2. DR	Patients with retinopathy exhibited levels of Lp(a) similar to those of patients without retinopathy.
Ginier et al., 1997 [38]	Cross-sectional	95	61.7 ± 1.4	100%	1. DM and NO DR 2. DR	Patients with retinopathy had higher Lp(a) concentrations than those without retinopathy.
Lip et al., 1998 [39]	Cross-sectional	21	67 ± 10.6	57.1%	1. NO DM + NO DR 2. NPDR 3. PDR	Patients with DR exhibited higher Lp(a) levels than those without DR.
Hadjadj et al., 2004 [40]	Cohort	297	33.8 ± 11.2	58.5%	1. DM and NO DR 2. NPDR 3. SEVERE NPDR 4. PDR	Lp(a) levels were higher when diabetic complications were more severe.
Asakawa et al., 2000 [41]	Cross-sectional	150	60.3 ± 11.0	52.7%	1. DM and NO DR 2. DR	Lp(a) is not a risk factor for microangiopathy and microvascular complications such as retinopathy.
Maser et al., 1993 [42]	Cross-sectional	186	34 ± 8	N/A	1. DM and NO DR 2. PDR	Lp(a) concentrations were not significantly different for those with or without proliferative retinopathy.

Lp(a)—Lipoprotein(a); DM—Diabetes Mellitus; DR—Diabetic Retinopathy; NPDR—Non-Proliferative Diabetic Retinopathy; PDR—Proliferative Diabetic Retinopathy; N/A—Not Available; yrs—years.

**Table 2:** Pathogenetic mechanisms of Lp(a) in Diabetic Retinopathy.

Pathogenetic Mechanisms	Details
Endothelial Dysfunction	Lp(a) reduces nitric oxide bioavailability, increases ROS quantities, and damages endothelial cells, disrupting the blood–retinal barrier.
Oxidative Stress	Induces ROS production in endothelial and retinal cells, damages mitochondria, and disrupts cellular metabolism.
Inflammation	Stimulates IL-6, IL-1β, TNF-α, ICAM-1, and VCAM-1 expression; promotes leukostasis and chronic inflammation.
Prothrombotic and Vasoconstrictive Effects	Mimics plasminogen, leading to prothrombotic activity and vasoconstriction, worsening retinal ischemia.
VEGF Upregulation and Neovascularization	Increases VEGF expression due to oxidative stress, driving pathological angiogenesis and retinal hemorrhages.
Extracellular Matrix Remodeling and MMP Activation	Enhances deposition of ECM proteins and activates MMPs, promoting angiogenesis and vascular remodeling.
Retinal Vascular Permeability and Pericyte Loss	Increases retinal vascular permeability and pericyte dropout, contributing to structural retinal damage.

ROS: Reactive oxygen species, VEGF: Vascular endothelial growth factor, MMP: Matrix metalloproteinases, ECM: extracellular matrix, IL-6: Interleukin-6, IL-1β: Interleukin-1 beta, TNF-α: Tumor Necrosis Factor-alpha, ICAM-1: Intercellular Adhesion Molecule-1, and VCAM-1: Vascular Cell Adhesion Molecule-1.

**Table 3:** Targeted Lp(a)-lowering therapeutics in phases of clinical trials.

Agent	Mechanism	Lp(a) Reduction
Muvalaplin [72]	oral	Up to 65%
Lepodisiran [71]	subcutaneous	Up to 95%
Zerlasiran [70]	subcutaneous	Up to 95%
Olpasiran [69]	subcutaneous	Up to 95%
Pelacarsen [73]	subcutaneous	Up to 92%

## Data Availability

All data generated or analyzed during this study are included in this article. Further enquiries can be directed to the corresponding author.

## Supplementary Materials

The following supporting information can be downloaded at https://www.mdpi.com/article/10.3390/medicina61071137/s1. Supplementary Table S1: Quality assessment results obtained using the Newcastle–Ottawa Quality Assessment Scale (NOS) tool.
