# Supplementary material for: Lipoprotein (a) in the Development and Progression of Diabetic Retinopathy: A Systematic Review and Meta-Analysis
Source: Medicina (Kaunas). 2025 Jun 24;61(7):1137. doi: 10.3390/medicina61071137 (PMC12299466; doi:10.3390/medicina61071137)
Supplement: Supplementary file 1 [file medicina-61-01137-s001.zip › medicina-3638817-supplementary.pdf]

Supplementary Table S1. Quality assessment results with the Newcastle-Ottawa Quality Assessment Scale (NOS) tool.

Study	*Design*	*Selection*	*Comparability*	*Exposure/ Outcome*	*Score*	*Quality*
Deraz et al., 2021	case-control	***	*	***	7/9	high
Kurt et al., 2002	cross-sectional	****	*	**	7/10	high
Malhotra et al., 2014	cross-sectional	**	*	**	5/10	moderate
Singh et al., 2009	cross-sectional	***	*	**	5/10	moderate
Rudberg et al.,1995	case-control	***	**	**	7/9	high
Tu et al., 2017	cross-sectional	***	*	***	7/9	high
Moosaie et al., 2020	case-control	****	**	***	9/10	high
Yun et al., 2016	cohort	***	**	**	7/9	high
Malaguarnera et al., 2013	cross-sectional	***	*	**	7/10	high
Liu et al., 2022	case-control	****	*	***	8/9	high
Chandni et al., 2012	cross-sectional	***	**	**	7/10	high
Morisaki et al., 1994	cross-sectional	**	*	**	6/10	moderate
Haffner et al., 1995	cross-sectional	***	*	***	7/10	high
Ergün et al., 2004	cross-sectional	***	*	**	6/10	moderate
Chopra et al., 2007	cross-sectional	***	*	**	6/10	moderate
Onuma et al., 1994	cross-sectional	**	*	**	5/10	moderate
Willems et al., 1996	cross-sectional	**	*	***	6/10	moderate
Suehiro et al., 2002	cross-sectional	***	*	***	7/10	high
Verrotti et al., 1997	cross-sectional	***	*	***	7/10	high
Kim et al., 1998	cross-sectional	**	*	**	5/10	moderate
Ritter et al., 1993	cross-sectional	***	*	***	7/10	high
Boemi et al., 1997	cross-sectional	***	*	**	6/10	moderate
Chen et al., 2022	case-control	***	**	***	8/9	high
Gazzaruso et al., 1998	cross-sectional	***	*	**	6/10	moderate
Ginier et al., 1997	cross-sectional	***	*	***	7/10	high
Lip et al., 1998	cross-sectional	**	**	***	7/10	high
Hadjadj et al., 2004	cohort	***	**	***	8/9	high
Asakawa et al., 2000	cross-sectional	****	*	***	8/10	high
Maser et al., 1993	cross-sectional	***	*	***	7/10	high

Case-Control
Selection (max 4 stars), Comparability (max 2 stars), Exposure (max 3 stars), Score (max score 9)
Cross Sectional
Selection (max 5 stars, Comparability (max 2 stars), Outcome (max 3 stars), Score (max score 10)
Quality
low: 0-3, moderate: 4-6 and high: 7-9 (or 7-10)
